# Medical physics in *Current Oncology*

**DOI:** 10.3390/curroncol13020002

**Published:** 2006-04

**Authors:** J. Seuntjens

**Affiliations:** * Medical Physics, McGill University, Montreal, Quebec

Medical physics can be generally defined as a field in which applied physics techniques are used in medicine. Traditionally, medical physics deals chiefly with the use of ionizing or non-ionizing radiation in the diagnosis and treatment of disease. In radiation therapy, ionizing radiation is used to treat a wide variety of cancers through external-beam radiotherapy or brachytherapy. Medical physics research and development are essential to maintaining and improving the success of these treatments.

Whereas the roles of imaging and of therapy using radiation have traditionally been separate, and imaging has traditionally been limited to the diagnosis of disease, we are currently witnessing a radically increasing role for imaging during and after therapy. The role of imaging during radiation therapy is to verify and, if need be, adapt the treatment according to the findings. Imaging developments include not only conventional anatomically based imaging such as computed tomography, but also imaging technology that provides information about metabolic activity and molecular functions and roles. Hence, the integration of various types of co-registered imaging into the planning and adaptation of therapy is an imperative step toward further optimization of radiation therapy efficacy. Such developments, integrated with state-of-the-art capabilities in Monte Carlo dose-calculation technology and modulated treatment delivery, form the basis of anticipated advances in radiation therapy physics over the next few years.

The role of the Medical Physics subsection in *Current Oncology* is thus to provide reports and reviews of these exciting developments in image-guided adaptive radiation therapy. *Current Oncology* also publishes critical evaluation studies of the clinical implementation and impact of novel and clinically established technologies.

In recent years, intensity modulated radiation therapy (imrt) has made its way into clinical radiation therapy. The imrt technique makes use of a large number of modulated treatment beams, optimized to conform the radiation dose to the target volume, sparing healthy tissues as much as possible. Planning techniques for imrt make use of dose–volume constraints for the target volume and organs at risk. The better conformity that can be achieved using inversely optimized imrt treatment delivery has led to a reduction in the margins used to define target volumes.

It might be expected that patient motion attributable to physiologic processes and patient set-up errors and uncertainties compromise the planned conformity of imrt. In the paper by Ploquin *et al.* in this issue, the effect of set-up errors and uncertainties on conformity in the imrt treatment of oropharyngeal cancer is investigated in comparison with treatment by traditional three-dimensional conformal radiation therapy (3D-crt). Using treatment planning studies with a large number of modified treatment plans, the authors find—surprisingly—that for the specified treatment site imrt maintains its superiority over 3D-crt despite the fact that imrt was found to be more sensitive to simulated set-up errors.

For different treatment sites, studies such as these may well reveal that set-up errors and uncertainties introduce problems by that compromise the advantage imrt holds over 3D-crt; however, more work will be needed to rigorously establish results.

